# Myeloperoxidase-Anti-Neutrophil Cytoplasmic Antibody Vasculitis With Isolated Renal Presentation in an Older Female Patient: A Diagnostic and Therapeutic Challenge

**DOI:** 10.14740/jmc5409

**Published:** 2026-10-02

**Authors:** Abrar Ahmad Zulfiqar, Alexandre Sebaux

**Affiliations:** a Geriatric Short-Stay Unit, Fecamp Hospital, Fecamp 76400, France; b Plancoet General Practice, Plancoet 22130, France

**Keywords:** ANCA-associated vasculitis, Older patient, PEXIVAS trial, MAINRITSAN trial

## Abstract

Anti-neutrophil cytoplasmic antibody (ANCA)-associated vasculitides are rare systemic autoimmune diseases, the incidence of which increases in older individuals. We report the case of a 76-year-old woman whose routine laboratory workup revealed acute kidney injury with a serum creatinine level of 155 µmol/L and significant proteinuria (2.5 g/24 h). ANCA testing was strongly positive for anti-myeloperoxidase (MPO) antibodies. A renal biopsy confirmed pauci-immune glomerulonephritis with moderate interstitial fibrosis and glomerulosclerosis, consistent with ANCA-associated vasculitis. Induction therapy consisted of corticosteroids and rituximab, which was discontinued following a suspected drug-related cutaneous reaction. Cyclophosphamide was successfully used as an alternative induction treatment. Following negative allergological evaluation and cautious re-exposure, rituximab was subsequently resumed for maintenance therapy and was well tolerated. Late-onset MPO-ANCA-associated vasculitis can have a favorable prognosis when diagnosed early and managed with appropriate immunosuppressive regimens.

## Introduction

Anti-neutrophil cytoplasmic antibody (ANCA)-associated vasculitides comprise a heterogeneous group of systemic autoimmune diseases characterized by necrotizing inflammation predominantly affecting small blood vessels [1, 2]. Although uncommon, their incidence increases with age. Among these conditions, myeloperoxidase (MPO)-ANCA-associated disease, typically encountered in microscopic polyangiitis (MPA), is particularly frequent in older adults. A study from the Anglo-Saxon countries [3] showed that the average age at diagnosis for MPA is approximately from 65 to 70 years, and that more than 60% of cases occur after the age of 65. In individuals over 75 years of age, MPA is the most common form of ANCA-associated vasculitis, surpassing granulomatosis with polyangiitis (GPA).

In older adults, the clinical presentation is often atypical, dominated by isolated renal involvement, with a clinical picture that can sometimes be misleading. The diagnostic process is therefore challenging due to overlap with conditions commonly seen in older adults, and management must incorporate the principles of geriatric medicine. Early diagnosis and appropriate immunosuppressive therapy can help limit renal sequelae. For active ANCA-associated vasculitis with organ-threatening manifestations, current recommendations support remission-induction therapy with glucocorticoids combined with rituximab or cyclophosphamide, rather than glucocorticoid monotherapy [1, 4].

We present here the case of an older female patient who developed pauci-immune anti-MPO-ANCA vasculitis with an exclusively renal presentation, illustrating the diagnostic and therapeutic characteristics of these late-onset forms.

## Case Report

A 76-year-old female patient has a long history of being treated for high blood pressure, high cholesterol, hypothyroidism (undergoing hormone replacement therapy), and irritable bowel syndrome. She lives at home with her husband, is independent in activities of daily living, and has no cognitive impairment, malnutrition, or sensory impairment. No relevant occupational or environmental exposure was identified, including exposure to silica, mineral or agricultural dust, or glass-industry activities. She consulted her primary care physician regarding recurrent episodes of urinary tract infections, specifically cystitis. Following her third episode of urinary tract infection, laboratory tests were performed. The results revealed renal insufficiency with a serum creatinine level of 155 µmol/L (normal: 44–71 µmol/L) (compared to a previously documented value of 70 µmol/L that was performed 3 months earlier, and corresponding to an epidermal glomerular filtration rate (eGFR) of 73 mL/min/1.73 m^2^). The decline in renal function is therefore recent and significant. Hemoglobin is low at 9.2 g/dL, and hyponatremia is noted at 132 mmol/L (normal: 135–145 mmol/L).

Urinalysis shows proteinuria at 2.5 g/24 h (normal < 0.14 g/24 h) with microalbuminuria at 220 mg/L. Protein electrophoresis reveals no monoclonal abnormalities; Bence-Jones proteins are negative. ANCA testing is positive with a titer of 160 (normal < 20) of the perinuclear (p)-ANCA type, confirmed by indirect immunofluorescence. Anti-MPO antibodies are strongly positive (> 8.0 (antibody index (AI)), while anti-proteinase 3 (PR3) antibodies are negative. Other antinuclear antibodies (ANAs) are negative. The patient’s laboratory results at admission are summarized in Table 1.

**Table 1:** Key Laboratory Findings at Admission

Parameters	Values	Reference ranges
Hemoglobin	9.2 g/dL	11.8–15.0 g/dL
Sodium	132 mmol/L	135–145 mmol/L
Serum creatinine	155 µmol/L	44–71 µmol/L
eGFR (CKD-EPI)	28 mL/min/1.73 m^2^	> 90 mL/min/1.73 m^2^
Serum uric acid	74 mg/L	24–66 mg/L
NT-proBNP	588 pg/mL	< 125 pg/mL
CRP	< 4 mg/L	< 10 mg/L
24-h proteinuria	2.5 g/24 h	< 0.14 g/24 h
p-type ANCA (titer)	160 (< 20)	Negative
Anti-MPO	> 8.0 AI	< 1.1 AI
Microalbuminuria	220 mg/L	< 30 mg/L

eGFR (CKD-EPI): epidermal glomerular filtration rate (chronic kidney disease epidemiology) collaboration; NT-proBNP: N-terminal proBNP; anti-MPO: anti-myeloperoxidase; p-type ANCA: perinuclear-type anti-neutrophil cytoplasmic antibody; CRP: C-reactive protein; AI: antibody index.

Abdominal-pelvic ultrasound and thoraco-abdominal-pelvic computed tomography (CT) scan revealed no urinary obstruction or extrarenal involvement. The patient was admitted to the Nephrology unit for further evaluation, including a renal biopsy.

The renal biopsy, performed after correcting a hemoglobin level of 7 g/dL (normal: 11.8–15.0 g/dL) via transfusion of two units of packed red blood cells (PRBCs), showed nine glomeruli, four of which were obliterated (segmental glomerulosclerosis), ischemic lesions, and moderate interstitial fibrosis (10–20%). No active lesions or extracapillary proliferation were observed. Immunofluorescence was negative. The pauci-immune pattern, in the context of strong anti-MPO-ANCA positivity, led to a diagnosis of ANCA-associated vasculitis with exclusive renal involvement.

Induction therapy was initiated: corticosteroid therapy with bolus infusion of Solu-Medrol (Pfizer, New York, NY, USA) for 3 days, followed by oral maintenance therapy according to the plasma exchange and glucocorticoids for treatment of ANCA-associated vasculitis (PEXIVAS) protocol. Treatment with rituximab (375 mg/m^2^ weekly) was initiated, but 3 days after the first infusion, the patient developed a diffuse erythematous and pruritic cutaneous reaction following the concomitant initiation of trimethoprim–sulfamethoxazole (Bactrim). Rituximab was discontinued during induction, and treatment was switched to intravenous cyclophosphamide (500 mg every 2–3 weeks for six cycles). Subsequent allergological evaluation, including intradermal testing, was negative for rituximab, while the causality of trimethoprim–sulfamethoxazole remained uncertain. After multidisciplinary allergological assessment, rituximab was cautiously reintroduced using a fractionated re-introduction protocol, consisting of 1/10 of the rituximab dose on day 1 and the remaining dose on day 8, with premedication and a slower infusion rate, and close clinical monitoring. Re-exposure was well tolerated without recurrence of the cutaneous reaction, allowing rituximab to be subsequently used for maintenance therapy.

The clinical course was favorable, with serum creatinine decreasing from 155 to 116 µmol/L and 24-h proteinuria decreasing from 2.5 to 0.6 g/24 h. Maintenance therapy with rituximab was initiated every 6 months. The patient’s key laboratory findings are shown in Table 2, and her treatment history as her clinical and laboratory status evolved is detailed in Table 3.

**Table 2:** Key Laboratory Findings

Parameter	Initial value	Control value	Reference ranges
Serum creatinine	155 µmol/L	116 µmol/L	44–71 µmol/L
eGFR (CKD-EPI)	28 mL/min/1.73 m^2^	48 mL/min/1.73 m^2^	> 60 mL/min/1.73 m^2^
Hemoglobin	9.2 g/dL	> 12 g/dL	11.8–15.0 g/dL
Sodium	132 mmol/L	137 mmol/L	135–145 mmol/L
24-h proteinuria	2.5 g/24 h	0.6 g/24 h	< 0.14 g/24 h
Anti-MPO-ANCA	> 8.0 AI	Decrease	< 1.1 AI
CRP	< 4 mg/L	Negative	< 10 mg/L

eGFR (CKD-EPI): epidermal glomerular filtration rate (chronic kidney disease epidemiology) collaboration; anti-MPO-ANCA: anti-myeloperoxidase anti-neutrophil cytoplasmic antibody; CRP: C-reactive protein; AI: antibody index.

**Table 3:** Summary of Treatments Administered

Treatment	Main modalities	Comments
Corticosteroid therapy	Three bolus doses of methylprednisolone sodium succinate (Solu-Medrol), followed by oral therapy according to PEXIVAS	Planned gradual tapering
Rituximab	375 mg/m^2^ weekly × 4	Diffuse erythematous and pruritic reaction after the first infusion; temporarily discontinued and subsequently re-introduced after allergological assessment using a fractionated protocol, premedication, and a slower infusion rate
Cyclophosphamide	500 mg IV every 2–3 weeks × 6 cycles	Effective temporary replacement
Rituximab (maintenance)	500 mg every 6 months	Successfully re-introduced after allergological assessment and subsequently continued as maintenance therapy
Anti-infective prophylaxis	Atovaquone (Wellvone)	Substitute for trimethoprim–sulfamethoxazole (Bactrim)
Other measures	Vaccination, close monitoring	Good clinical stabilization

IV: intravenous; PEXIVAS: plasma exchange and glucocorticoids for treatment of anti-neutrophil cytoplasmic antibody-associated vasculitis.

## Discussion

ANCA-associated vasculitides in older adults exhibit clinical, laboratory, and histopathological characteristics that may differ from those observed in younger patients. Anti-MPO forms predominate in this population, whereas anti-PR3 forms are more common in younger adults [1]. Clinical presentation may be atypical, with renal-limited disease and fewer ear, nose and throat (ENT), pulmonary, or systemic manifestations [1, 2]. This may complicate diagnosis, as renal impairment can initially be attributed to age-related nephropathy or nephrosclerosis. Histologically, renal biopsies in older patients may show a higher proportion of glomerulosclerosis and interstitial fibrosis, reflecting both chronic vascular or degenerative changes and delayed recognition of the disease.

In our patient, the decision to initiate immunosuppressive therapy was based on an integrated assessment rather than renal histology alone. Despite the absence of active necrotizing lesions or extracapillary proliferation in the biopsy specimen, renal function had deteriorated rapidly, with serum creatinine increasing from 70 to 155 µmol/L within 3 months, accompanied by substantial proteinuria (2.5 g/24 h), a strongly positive p-ANCA titer, and markedly elevated anti-MPO antibodies. The biopsy contained only nine glomeruli and demonstrated a pauci-immune pattern with glomerulosclerosis and moderate interstitial fibrosis. In this clinical and immunological context, the absence of active lesions in the sampled glomeruli was not considered sufficient to exclude clinically active renal-limited ANCA-associated vasculitis. After multidisciplinary assessment of the expected benefits and treatment-related risks, induction immunosuppression was initiated with the aim of controlling the disease and preserving residual renal function.

Therefore, this case highlights the importance of integrating clinical evolution, renal function, urinary abnormalities, ANCA serology, and histopathological findings rather than relying on a single parameter. Renal biopsy nevertheless remains essential for establishing the diagnosis, assessing chronic damage, and guiding therapeutic decisions, particularly in older patients in whom competing causes of renal dysfunction are frequent.

Treatment of ANCA-associated vasculitis in older adults requires careful balancing of disease control against treatment-related toxicity. Current European Alliance of Associations for Rheumatology (EULAR) recommendations support glucocorticoids combined with rituximab or cyclophosphamide for remission induction [1, 4]. The PEXIVAS trial demonstrated that a reduced-dose glucocorticoid regimen was non-inferior to a standard-dose regimen for death or end-stage kidney disease and was associated with fewer serious infections during the first year [5]. These findings are particularly relevant in older patients, who are more susceptible to infectious, metabolic, muscular, and neuropsychiatric complications of prolonged corticosteroid exposure.

Rituximab and cyclophosphamide are both established options for remission induction, and treatment choice should be individualized according to comorbidities, infection risk, hematological tolerance, and treatment-related adverse events [1, 4]. Data specifically obtained in older populations also support the efficacy of both approaches. Aqeel et al reported high remission rates with cyclophosphamide, rituximab, or their combination in patients aged ≥ 60 years with renal involvement, although leukopenia was less frequent with rituximab alone [6]. Similarly, individualized regimens combining reduced immunosuppressive exposure have been proposed for frail older patients with severe ANCA-associated glomerulonephritis [7].

In our patient, rituximab was initially discontinued after a diffuse erythematous and pruritic cutaneous reaction occurring after the first infusion, leading to a temporary switch to cyclophosphamide. Subsequent allergological investigations did not confirm rituximab hypersensitivity, and cautious fractionated re-introduction with premedication, a slower infusion rate, and close monitoring were well tolerated. This illustrates the importance of specialist re-assessment of suspected drug hypersensitivity when therapeutic alternatives are limited.

For maintenance therapy, rituximab has demonstrated superiority to azathioprine in preventing major relapse in ANCA-associated vasculitis [8, 9]. Maintenance of remission using rituximab in systemic ANCA-associated vasculitis (MAINRITSAN)-derived data have also shown preservation of physical function compared with azathioprine [10], while pooled long-term analyses support sustained remission with rituximab-based maintenance strategies [11]. These findings supported the subsequent use of rituximab maintenance in our patient after successful re-exposure.

Beyond disease-specific therapy, geriatric factors should be incorporated into therapeutic decision-making. Frailty, comorbidities, polypharmacy, cognitive status, and infection risk may influence both treatment tolerance and prognosis. McGovern et al showed that advanced age, frailty assessed using the Rockwood approach [12], and elevated C-reactive protein (CRP) at diagnosis were associated with mortality in older patients with ANCA-associated vasculitis [13]. Comprehensive geriatric assessment may therefore complement nephrological and immunological evaluation when determining treatment intensity.

In our patient, preserved functional status allowed for standard disease-directed treatment with close multidisciplinary follow-up involving nephrology, allergology, and primary care. Atovaquone (Wellvone, GlaxoSmithKline, London, UK) provided an alternative for anti-infective prophylaxis when trimethoprim–sulfamethoxazole remained contraindicated. Despite the presence of chronic histological lesions, partial renal recovery was achieved, with serum creatinine decreasing from 155 to 116 µmol/L and proteinuria from 2.5 to 0.6 g/24 h. This favorable evolution supports an individualized therapeutic approach based on the overall clinical, biological, histological, and geriatric profile rather than chronological age or renal histology alone.

### Conclusions

This clinical case illustrates the complexity of diagnosing and managing late-onset ANCA-associated vasculitis in older adults. The atypical clinical presentation, isolated renal involvement, histological features, and specific therapeutic challenges warrant an integrated approach. The use of validated protocols and multidisciplinary evaluation remains key to a favorable prognosis.

### Learning points

Late-onset MPO-ANCA-associated vasculitis may present as isolated renal disease without typical systemic manifestations, making early recognition challenging in older adults and emphasizing the importance of maintaining a high index of suspicion in cases of unexplained acute kidney injury.

Renal biopsy remains essential for confirming the diagnosis of pauci-immune glomerulonephritis and should not be delayed in older patients, even when chronic renal lesions predominate, as timely immunosuppressive therapy may preserve kidney function.

Management of ANCA-associated vasculitis in older adults should be individualized, and alternative induction strategies such as cyclophosphamide may be successfully used when rituximab is contraindicated or poorly tolerated, followed by appropriate maintenance therapy and multidisciplinary follow-up.

## Data Availability

All relevant data supporting the findings of this case report are included in the article.
